# Shorter leukocyte telomere length protects against NAFLD progression in children

**DOI:** 10.1038/s41598-023-31149-y

**Published:** 2023-04-03

**Authors:** Janet M. Wojcicki, Ryan M. Gill, Laura Wilson, Jue Lin, Philip Rosenthal

**Affiliations:** 1 Division of Pediatric Gastroenterology, Hepatology and Nutrition, University of California, San Francisco, CA USA; 2 Department of Epidemiology and Biostatistics, University of California, San Francisco, CA USA; 3 Department of Pathology, University of California, San Francisco, CA USA; 4 Department of Epidemiology, Bloomberg School of Public Health, Johns Hopkins University, Baltimore, MD USA; 5 Department of Medicine, School of Medicine, Johns Hopkins University, Baltimore, MD USA; 6 Department of Biochemistry and Biophysics, University of California, San Francisco, CA USA

**Keywords:** Genetics, Gastroenterology, Molecular medicine

## Abstract

Leukocyte telomere length (LTL) gets shorter with each cell division and is also sensitive to reactive oxygen species damage and inflammatory processes. Studies in adults with non-alcoholic fatty liver disease (NAFLD) have found that increased fibrosis but not ALT levels are associated with shorter LTL. Few pediatric studies have been conducted; as such, we sought to evaluate potential associations between LTL and liver disease and liver disease progression in pediatric patients. Using data from the Treatment of NAFLD in Children (TONIC) randomized controlled trial, we assessed the potential predictive relationship between LTL and liver disease progression based on two successive liver biopsies over 96 weeks. We assessed the potential relationship between LTL and child age, sex, and race/ethnicity and features of liver disease including components of histology. We subsequently evaluated predictors for improvement in non-alcoholic steatohepatitis (NASH) at 96 weeks including LTL. We also assessed predictors of lobular inflammation improvement at 96 weeks using multivariable models. Mean LTL at baseline was 1.33 ± 0.23 T/S. Increasing lobular and portal inflammation were associated with longer LTL. In multivariable models, greater lobular inflammation at baseline was associated with longer LTL (Coeff 0.03, 95% CI 0.006–0.13; p = 0.03). Longer LTL at baseline was associated with worsening lobular inflammation at 96 weeks (Coeff 2.41, 95% CI 0.78–4.04; p < 0.01). There was no association between liver fibrosis and LTL. The association between LTL and pediatric NASH does not parallel adults with no association between fibrosis stage and NASH. Conversely, longer LTL was associated with more lobular inflammation at baseline and increased lobular inflammation over the 96-week period. Longer LTL in children may indicate greater risk for future complications from NASH.

## Introduction

Non-alcoholic fatty liver disease (NAFLD) encompasses a spectrum of fatty liver change, including non-alcoholic steatohepatitis (NASH), that can be considered to represent a hepatic manifestation of metabolic syndrome. Obesity, specifically abdominal obesity, and insulin resistance increase risk for NAFLD and NASH in children. NASH is defined by presence of > 5% macrovesicular steatosis, hepatocyte ballooning, and lobular inflammation and typically also has portal lymphocytic inflammation. This typical (type 1) pattern is observed in most adults, whereas in children an alternate (type 2) pattern, consisting of severe panacinar or periportal macrovesicular steatosis and portal-based fibrosis and inflammation is recognized. NALFD is an umbrella term that includes NASH and non-alcoholic fatty liver (NAFL), which has much lower risk for progression to liver fibrosis, cirrhosis, and eventual liver transplantation than NASH, though NAFL with lobular inflammation may confer some increased risk of fibrosis over NAFL without lobular inflammation^1^.

Factors associated with fibrosis progression to cirrhosis and from NAFL to NASH are not well delineated, particularly in pediatric patients. Furthermore, NASH in children may manifest with a type 1 or type 2 pattern, or show transition from one type to another (typically from type 2 to type 1), particularly in older children and adolescents^2^. More severe steatosis is often seen in pediatric NAFLD patients compared with adults^3^. Patches of microvesicular steatosis have been associated with more severe hepatocyte injury including a diagnosis of NASH or probable NASH based on adult studies^4^ but these associations have yet to be characterized in pediatric NAFLD patients.

Recent studies have focused on the potential role of telomeres, the non-coding DNA sequences that protect chromosomes from damage and degradation, specifically hepatocyte telomeres as playing a potential role in the process of liver aging that is associated with NAFLD, specifically NASH and fibrosis processes^5^. It is suggested that repeated antigen exposure can impact telomerase up-regulation as could exposure to oxidative stress which would trigger p21 upregulation and cellular apoptosis^6^. In adults with NASH, shorter leukocyte and hepatocyte telomere length is associated with more advanced fibrosis^5,7^. In the healthy aging liver, hepatocyte and cholangiocyte telomere length is preserved compared with Kupffer and stellate cells where telomere shortening occurs^8^. By contrast, telomere shortening processes are observed in cirrhosis in many different liver cell types^9^. Other studies including some of our own suggest that leukocyte telomere length may also be associated with liver disease and fibrosis in the context of NAFLD and NASH in adults^7,10,11^. Few studies, however, have assessed these relationships in pediatric populations.

Using data collected from the National Institute of Diabetes and Digestive and Kidney Diseases sponsored Treatment of Non-alcoholic Fatty Liver Disease in Children Trial (TONIC) (NCT00063635), we evaluated the relationship between leukocyte telomere length (LTL) in children with NAFLD and NASH in TONIC. We additionally assessed the potential role of LTL as a predictive biomarker of NASH progression and regression using data points from this trial over 96 weeks of follow-up. As there was detailed liver biopsy characteristics on each child at two time points, we sought to understand the role that LTL might be play to help elucidate the process of NAFLD progression in children.

## Methods

The Treatment of NAFLD in Children Trial (TONIC) was a NIH sponsored randomized, double-blind, placebo-controlled trial that was conducted at 10 research centers in the US with 173 patients (age 8–17) with biopsy confirmed NAFLD to assess the impact of vitamin E (400 IU twice daily) compared with 500 mg metformin twice daily versus placebo over 96 weeks (ClinicalTrials.gov identifier: NCT00063635, 03/07/2003). The primary outcome was reduction in serum alanine aminotransferase (ALT) levels^12^ and has been previously described in protocol and result publications including full CONSORT checklist^13,14^. Different study sites each had the respective institutional review board approve the study. Informed consent was obtained from all children (subjects) and/or their legal guardians. For this secondary data analysis of study results, the University of California, San Francisco (UCSF) institutional review board (IRB) provided approval for this study and all guidelines and regulations were followed as indicated by the IRB.

As part of the TONIC trial, whole blood was collected from children to collect DNA samples for potential genetic testing. DNA was extracted using the Qiagen Autopure LS DNA extractors using PUREGENE reagents using a modified salting out procedure. DNA was rehydrated by incubating at 56 °C for 1 h in a shaking incubator and placed on orbital shakers for 36–48 h prior to stock transfer. In the event a sample had a 260/280 ratio less than 1.7 (indicative of protein contamination), it was re-precipitated to improve its purity. DNA extraction was performed as soon as samples were received by the lab. Samples that were processed for DNA extraction and passed quality control were assigned storage locations by RUCDR STARLIMS in a − 80 °C freezer until LTL assay.

Liver biopsies were obtained at study entry and subsequently after 96 weeks of therapy for all children enrolled in the TONIC trial. For all biopsies, the NASH Clinical Research Network (CRN) recommended a 16-gauge biopsy needle and a specimen length of at least 1.5 cm. The liver tissue was prepared locally for light microscopy with stains including hematoxylin and eosin, Masson’s trichrome and iron stain. All slides were sent to the data coordinating center for central reading by the study pathologists^15^. Slides were assessed for steatohepatitis, fibrosis and NAFLD Activity Score (NAS) (consisting of steatosis, lobular inflammation, and hepatocyte ballooning) using previously defined criteria^16^. Other specifics that were extracted from the biopsy included factors associated with metabolic disease and obesity (e.g., megamitochondria, glycogen nuclei, large lipogranulomas, microvesicular steatosis) and others associated with cell death and hepatic injury (e.g., acidophil bodies, Mallory bodies, microgranulomas, and pigmented macrophages).

At baseline and 96 weeks of follow-up liver function tests and biomarkers of metabolic health including lipids and insulin resistance were also evaluated in the children. Anthropometrics including body mass index (BMI) and waist circumference were also assessed. Additionally, at baseline, autoantibodies associated with autoimmune hepatitis were assessed including anti-nuclear antibody (ANA), anti-smooth muscle antibody (ASMA) and anti-mitochondrial antibody (AMA).

### Leukocyte telomere length analysis

Extracted DNA was shipped in batch to the Blackburn Telomere Lab at the University of California, San Francisco. The telomere qPCR primers were tel1b [5′-CGGTTT(GTTTGG)5GTT-3′], used at a final concentration of 100 nM, and tel2b [5′-GGCTTG(CCTTAC)5CCT-3′], used at a final concentration of 900 nM. The single-copy gene (human beta-globin) qPCR primers were hbg1 [5′-GCTTCTGACACAACTGTGTTCACTAGC-3′], used at a final concentration of 300 nM, and hbg2 [5′-CACCAACTTCATCCACGTTCACC-3′], used at a final concentration of 700 nM. The final reaction mix consisted of the following: 20 mM Tris-hydrochloride, pH 8.4; 50 mM potassium chloride; 200 μM each deoxyribonucleotide triphosphate; 1% dimethyl sulfoxide; 0.4 × SYBR green I; 22 ng *Escherichia coli* DNA; 0.4 Units of platinum Taq DNA polymerase (Invitrogen Inc., Carlsbad, CA), and approximately 6.6 ng of genomic DNA per 11 µl reaction. A threefold serial dilution of a commercial human genomic DNA (Sigma-Aldrich, cat#11691112001) containing 26, 8.75, 2.9, 0.97, 0.324 and 0.108 ng of DNA was included in each PCR run as the reference standard. The quantity of targeted templates in each sample was determined relative to the reference DNA sample by the maximum second derivative method in the Roche LC480 program. The reaction was carried out in a Roche LightCycler 480 in 384-well plates, with triplicate wells for each sample. Dixon Q test was used to exclude outliers from the triplicates. The average of the T and S triplicate wells after outlier removal was used to calculate the T/S ratio for each sample. The same reference DNA was used for all PCR runs.

The thermal profile for telomere (T) consisted of denaturing at 96 °C for 1 min followed by 30 cycles of denaturing at 96 °C for 1 s and annealing or extension at 54 °C for 60 s with fluorescence data collection. The thermal profile for a single copy gene (S) consisted of denaturing at 96 °C for 1 min followed by 8 cycles of denaturing at 95 °C for 15 s, annealing at 58 °C for 1 s, and extension at 72 °C for 20 s, followed by 35 cycles of denaturing at 96 °C for 1 s, annealing at 58 °C for 1 s, extension at 72 °C for 20 s, and holding at 83 °C for 5 s with data collection. The T/S ratio for each sample was measured in duplicate runs, each with triplicate wells. When the duplicate T/S values disagreed by more than 7%, the sample was run in triplicate and the two closest values were used. The Dixon Q test was used to remove outliers from the triplicates. The average of the T or S concentrations from the remaining wells was used to calculate the T/S ratio for each sample. This is repeated to get the run 2 T/S ratios. If the difference between run 1 and run 2 larger than 7%, a third run was performed and the average of the 2 closest values was used as the final data. Potential batch difference was adjusted by running combined plates with a small set of samples from each plate. All samples used the same lot of reagents for LTL assay. The average inter-assay coefficient of variation (CV) for this study was 2.2%. The PCR efficiencies were 96.2 ± 1.6% and 98.0 ± 2.9% for the telomere reactions (T) and S reactions (S) respectively. All samples were processed in the Blackburn Telomere Lab at UCSF. We did not calculate the interclass correlation coefficient (ICC) on repeat DNA extractions for this study because they were assayed before this recommendation was made by the Telomere Research Network.

### Data analysis

Data were checked for normality using graphical indicators as well as statistical tests such as Shapiro–Wilk. Baseline descriptive data were calculated including means ± SD and percentages of categorical data for patient demographics including age at DNA collection, ethnicity, sex, specifics regarding liver histology at baseline including steatosis, fibrosis, and components of NASH [ballooning, inflammation (lobular and portal)) as well as the NAFLD activity score (NAS) which incorporates lobular inflammation, ballooning, and steatosis]^16,17^. Other descriptive baseline data included liver function tests (LFTs) and other hepatic specific laboratory tests (bilirubin, alkaline phosphatase), anthropometrics (BMI and waist circumference), measures of insulin resistance and lipids (HDL, LDL, triglycerides, and total cholesterol).

We subsequently assessed what were potential predictive factors for NASH improvement, also defined as resolution of NASH, at 96 weeks, defining improvement as changing from definite or borderline NASH to not NASH based on biopsy alone. Other analyses included factors associated with LTL based at time of collection as an outcome. In most cases, the baseline data collection timepoint was most closely related to the LTL measurement including baseline biopsy, liver and fasting labs and anthropometric measurements. However, as not all patients had the DNA blood draw collected at the same time point in the study, in some cases biopsy or blood collection at the 96-week time point was used instead. As LTL data were not normally distributed, Mann Whitney U test was used for continuous predictors and chi-squared tests for categorical ones. Residuals of linear models were checked for normality using graphical tests and q-norm and p-norm plots. As the departures from normality were not severe, linear regression was used in multivariable models with LTL as the dependent variable.

Multivariable models were constructed to assess independent predictors for NASH improvement at 96 weeks (logistic regression) including variables that were significant at p < 0.10 in bivariate analysis (defined as moving from indeterminate NASH or definite NASH to not NASH), treatment group assignment (placebo versus metformin versus vitamin E) as well as LTL given our initial hypotheses and other variables with previously demonstrated biological plausibility for association with NASH improvement. We did not include collinear variables in multivariable models such as waist circumference and body mass index (BMI) simultaneously. We also constructed linear regression models to assess factors associated with longer leukocyte telomere at baseline (or at closest timepoint to when DNA was collected) including variables that were significant at p < 0.10 in bivariate analysis as well as those with biological plausibility based on previously determined biological plausibility with LTL.

Additional multivariable models were constructed to assess independent predictors for worsening of lobular inflammation using ordinal models (with lobular inflammation defined as 0 foci, less than 2 foci with 20 times magnification, 2–4 foci with 20 times magnification and greater than 4 foci with 20 times magnification) and the ordinal sequence being worsening, staying the same and improving from baseline to follow-up biopsy using the criteria as described above. Other multivariable models that we evaluated included changes in portal inflammation (none, mild, more than mild), fibrosis (none, mild Z3 perisinusoidal, moderate Z3 perisinusoidal, portal/periportal, Z3 periportal, bridging, cirrhosis) and ballooning (none, few, many) to try to test the hypothesis that LTL may be a biomarker to predict potential changes in hepatic health. These areas of hepatic histology were chosen given their importance in diagnosis of NASH and due to univariate findings suggesting associations between LTL and lobular and portal inflammation. Our criteria for constructing these models were similar in that we included predictors that were significant in bivariate analysis at p < 0.10 and others that were associated with biological plausibility as well as treatment assignment group for the TONIC trial.

Lastly, we evaluated other specific hepatic histology characteristics in relation to worsening in lobular inflammation including location of steatosis [Zone 3 (central), Zone 1 (periportal), Azonal, Panacinar] and type of steatosis microvesicular steatosis; markers of hepatic injury or necrosis such as acidophil bodies, Mallory bodies, microgranulomas and pigmented macrophages; and metabolic associated characteristics such as megamitochondria, glycogen nuclei and large lipogranulomas. Components of the NAS score including fibrosis stage (mild Z3 perisinusoidal, moderate Z3 perisinusoidal, portal/periportal, Z3 periportal or bridging) and ballooning were also assessed in relation to overall changing patterns of lobular inflammation.

## Results

### Population characteristics

In the TONIC trial, there were 149 children that had DNA samples collected with sufficient DNA to assess for child’s LTL. LTL measured 1.33 + /0.23 T/S units for the group as a whole (Table 1). The 149 children were randomized to three different experimental groups: metformin (34.2%), placebo (66.4%) and vitamin E (33.6%). Approximate age at LTL collection was 13.2 ± 2.5 years with 9.4% of the sample below the age of 10 (Table 1). More than half of the sample cited Mexican origin (59.7%) and 80.5% of patients were male. On baseline biopsy, 28.4% of patients had portal/periportal fibrosis with 21.0% having Z3 periportal and 23.7% having no fibrosis. Other significant findings on biopsy at baseline included the high percentage with some ballooning (few or many) (57.7%), 45% with > 66% steatosis, 41.6% with < 2 foci of lobular inflammation and 50.3% with 2–4 foci (with 20× magnification). Most of the sample was defined as having definite NASH or borderline NASH at baseline (83.2%) with mean NAFLD Activity Score (NAS) 4.58 ± 1.49 and mean ALT 120.9 ± 65.1 U/L (Table 1). A high percentage had microgranulomas (94.6%) and pigmented macrophages on biopsy (96.6%). Metabolic labs and anthropometrics at baseline included body mass index (BMI) (mean 33.9 ± 5.95 kg/m^2^), waist circumference (mean 108.2 ± 14.7 cm), fasting glucose (mean 89.1 ± 9.6 mg/dL), triglycerides (149.2 ± 96.6 mg/dL) and total cholesterol (173.3 ± 40.2 mg/dL).

**Table 1:** TONIC patients with leukocyte telomere length data (n = 149).

Variable	Number	Percentage/mean (SD)
Leukocyte telomere length (T/S ratio)		1.33 ± 0.23 (median 1.32 ± 0.23)
TONIC treatment group		
Metformin	51/149	34.2%
Placebo	48/149	66.4%
Vitamin E	30/149	33.6%
Patient demographics		
Age at telomere collection (years)	13.2 ± 2.5	
Age category (years)		
< 10	14/149	9.4%
10–13	58/149	38.9%
> 13	77/149	51.2%
Mexican origin	86/144	59.7%
Sex at birth		
Female	29/149	19.5%
Male	120/149	80.5%
Liver health at baseline		
General liver biopsy findings		
Fibrosis (baseline)		
None	35/148	23.7%
Mild Z3	11/148	7.4%
Moderate Z3	9/148	6.1%
Portal/periportal	42/148	28.4%
Z3 periportal	31/148	21.0%
Bridging	20/148	13.5%
Ballooning (baseline)		
Zero	63/149	42.3%
Few	52/149	34.9%
Many	34/149	22.8%
Steatosis (baseline)		
0. 0–5%	3/149	2.0%
1. > 5–33%	41/149	27.5%
2. > 33–66%	38/149	25.5%
3. > 66%	67/149	45.0%
Steatosis baseline (location)		
Zone 1/periportal	17/149	11.4%
Zone 3/central	39/149	26.2%
Azonal	16/149	10.7%
Panacinar	77/149	51.7%
Lobular inflammation (baseline) (20× magnification)		
0	1/149	0.7%
< 2	62/149	41.6%
2–4	75/149	50.3%
> 4	11/149	7.4%
Portal inflammation (baseline)		
None	11/149	7.4%
Mild	120/149	80.5%
More than mild	18/149	12.1%
NASH diagnosis at baseline		
Not NASH	25/149	16.8%
Z3 borderline	27/149	18.1%
Z1 borderline	36/149	24.2%
Definite NASH	61/149	40.9%
NASH activity score (NAS) (lobular + ballooning + steatosis)	4.58 ± 1.49	
Hepatic injury/necrosis specific parameters from liver biopsy (baseline)		
Acidophil bodies		
None/rare	73/149	49.0%
Many	76/149	51.0%
Mallory bodies		
None/rare	145/149	97.3%
Many	4/149	2.7%
Microgranuloma		
None/rare	8/149	5.4%
Many	141/149	94.6%
Pigmented macrophages		
None/rare	5/149	3.4%
Many	144/149	96.6%
Metabolic-associated characteristics from liver biopsy (baseline)		
Glycogen nuclei		
None/rare	74/149	49.7%
Many	75/149	50.3%
Large lipogranuloma		
None/rare	116/149	77.9%
Many	33/149	22.0%
Megamitochondria		
None/rare	135/149	90.6%
Many	14/149	9.4%
Microvesicular steatosis		
None/rare	138/149	92.6%
Many	11/149	7.4%
Liver function tests and auto-antibodies		
Alanine transaminase (ALT), U/L		
ALT (baseline)	120.9 ± 65.1	
ALT (96 weeks)	83.4 ± 62.8	
ALT change	− 37.8 ± 72.1	
Aspartate transaminase (AST), U/L		
AST (baseline)	69.9 ± 41.5	
AST (96 weeks)	51.5 ± 34.4	
AST change	19.2 ± 42.4	
Alkaline phosphatase (U/L)		
Alkaline phosphatase (baseline)	223.4 ± 94.7	
Alkaline phosphatase (96 weeks)	168.2 ± 88.4	
Alkaline phosphatase change	55.9 ± 70.2	
Bilirubin (mg/dL)		
Total (baseline)	0.67 ± 0.30	
Total (96 weeks)	0.73 ± 0.40	
Total change	− 0.06 ± 0.31	
Direct (baseline)	0.10 ± 0.07	
Direct (96 weeks)	0.11 ± 0.08	
Direct change	− 0.003 ± 0.06	
Auto-antibodies		
Anti-nuclear antibody (ANA positive)	31/149	20.9%
Anti-smooth muscle Antibody (ASMA positive)	56/149	37.6%
Anti-mitochondrial (AMA) antibody	1/149	0.7%
Metabolic health		
Anthropometrics		
Body mass index (BMI) (kg/m^2^)		
Baseline	33.9 ± 6.0	
96 weeks follow-up	35.5 ± 6.3	
BMI change (baseline to follow-up)	1.85 ± 3.0	
Waist circumference (cm)		
Baseline	108.2 ± 14.7	
96 weeks (follow-up)	113.2 ± 15.0	
Waist change (cm)	5.2 ± 9.1	
Systolic blood pressure (mmHg)	125.0 ± 15.5	
Diastolic blood pressure (mmHg)	69.4 ± 11.2	
Glucose control		
Glucose (fasting), mg/dL	89.1 ± 9.6	
Insulin (fasting), mcU/mL	42.3 ± 50.4	
Glucose tolerance (OGTT)		
Test 2 h (mg/dL)	119.8 ± 25.8	
HOMA-IR	9.03 ± 11.9	
≥ 3.99 ml/DL	117/149	78.5%
< 3.99 ml/DL	32/149	21.5%
Lipids		
LDL (mg/dL)	107.0 ± 30.6	
HDL (mg/dL)	37.0 ± 7.9	
Triglycerides (mg/dL)	149.2 ± 96.6	
Total cholesterol (mg/dL)	173.3 ± 40.2	

### Factors associated with NASH improvement over 96 weeks

There were 47 (35.1%) children who improved during the follow-up period and 87 who did not (64.0%) (Table 2). Patients in the metformin and Vitamin E groups improved more often than those in the placebo group (30.4% and 51.1% versus 22.0%; p = 0.01; Table 2). Those children that improved tended to be younger in age (12.65 ± 2.00 years versus 13.42 ± 2.82 years) although the results were not statistically significant (p = 0.10 Table 2). Those that improved also were less likely to have lobular inflammation, but results were also not statistically significant (p = 0.17) and steatosis most often in Zone 1 (50%) or panacinar (42%) (Table 2). Those that improved also had more hepatocyte ballooning at baseline (p = 0.05), and those that improved tended to have less microvesicular steatosis (37.7% absent versus 8.3% present, p = 0.04; Table 2).

**Table 2:** NASH improvement* from baseline to 96 weeks.

	Improve	Did not improve	p value
	47/134 (35.1%)	87/134 (64.0%)	
Leukocyte telomere length (T/S)	1.33 ± 0.22	1.34 ± 0.22	0.90
TONIC treatment group			
Metformin	14/46 (30.4%)	32/46 (64.6%)	
Vitamin E	24/47 (51.1%)	23/47 (48.9%)	
Placebo	9/41 (22.0%)	32/41 (78.1%)	0.01
Demographics			
Age (years)	12.65 + /2.00	13.42 ± 2.82	0.10
Mexican	31/45 (68.9%)	52/84 (61.9%)	0.43
Sex at birth			
Male	39/109 (35.8%)	70/109 (64.2%)	0.72
Female	8/25 (32.0%)	17/25 (68.0%)	
Liver health at baseline			
General liver biopsy findings			
Lobular inflammation			
1 (< 2)	20/53 (37.7)	35/53 (62.3)	0.17
2 (2–4)	26/70 (37.1)	44/70 (62.9)	
3 (> 4)	1/11 (9.1)	10/11 (90.1)	
Portal inflammation			
None	3/11 (27.3)	8/11 (72.7)	0.53
Mild	40/107 (37.4)	67/107 (62.6)	
More than mild	4/16 (25.0)	12/16 (75.0)	
Steatosis			
0—< 5%	0/1 (0.0)	1/1 (100)	0.26
1—5–33%	9/34 (26.5)	25/34 (73.5)	
2—> 33–66%	11/37(29.7)	26/37 (29.9)	
3—> 66%	27/62(43.6)	35/62 (56.5)	
Steatosis location baseline			0.05
Zone 1 (periportal)	8/16 (50.0)	8/16 (50.0)	
Zone 3 (central)	7/34 (20.6)	27/34 (79.4)	
Panacinar	29/69 (42.0)	40/69 (58.0)	
Azonal	3/15 (20.0)	12/15 (80.0)	
Ballooning			
None	15/57 (26.3)	42/57 (73.7)	0.05
Few	22/45 (48.9)	23/45 (51.1)	
Many	10/32 (31.3)	22/32 (68.8)	
NAFLD activity score (NAS score), baseline (steatosis + lobular + ballooning)	4.9 ± 1.3	4.6 ± 1.5	0.29
Hepatic injury/necrosis specific parameters from liver biopsy			
Acidophil bodies			
Rare/absent	23/67 (34.3)	44/67 (65.7)	0.86
Many	24/67 (35.8)	43/67 (64.2)	
Mallory bodies			0.67
Rare/absent	46/130 (35.4)	84/130 (64.6)	
Many	1/4 (25.0)	3/4 (75.0)	
Microgranulomas			
No	2/8 (25.0)	6/8 (75.0)	0.54
Yes	45/126 (35.7)	81/126 (64.3)	
Pigmented macrophages			
Rare/absent	1/4 (25.0)	3/ 4(75.0)	0.67
Many	46/130 (35.4)	84/130 (64.6)	
Metabolic associated characteristics from liver biopsy			
Megamitochondria			
Rare/absent	43/120 (35.8)	77/120 (64.2)	0.59
Many	4/14(28.6)	10/14 (71.4)	
Microvesicular steatosis			
Absent	46/122(37.7)	76/122 (62.3)	0.04
Present	1/12 (8.3)	11/12 (91.7)	
Glycogen nuclei			
Rare/absent	25/69 (36.2)	44/69 (63.8)	0.77
Many	22/65 (33.9)	43/65 (66.2)	
Large lipogranuloma			
No	39/105 (37.1)	66/105 (62.9)	0.34
Yes	8/29 (27.6)	21/29 (72.4)	
Liver function tests and auto-antibodies			
Liver enzymes (baseline)			
ALT (U/L)	111.8 ± 59.0	125.1 ± 68.7	0.29
AST (U/L)	64.8 ± 32.5	73.8 ± 47.0	0.63
Alkaline phosphatase (U/L)	238.9 ± 82.7	216.3 ± 100.4	0.08
Total bili (mg/dL)	0.7 ± 0.3	0.7 ± 0.3	0.39
Direct bill (mg/dL)	0.1 ± 0.1	0.1 ± 0.1	0.54
Auto-antibodies (liver)			
Anti-nuclear antibody (ANA)	11/29 (37.9)	18/29 (62.1)	0.72
Anti-smooth muscle			
Antibody (ASMA)	20/50 (40.0)	30/50 (60.0)	0.35
Anti-mitochondrial antibody	0/1 (0)	1/1 (100.0)	0.46
Metabolic health at baseline			
Blood pressure			
Systolic (mm Hg)	126.1 ± 15.6	125.0 ± 14.9	0.68
Diastolic (mm Hg)	67.94 ± 11.2	69.8 ± 11.6	0.40
Glucose control			
Fasting glucose (mg/dL)	86.8 ± 9.7	90.3 ± 9.3	0.04
Fasting insulin (mcU/mL)	36.4 ± 23.8	45.6 ± 60.5	0.32
Glucose tolerance (OGTT) test 2 h (mg/dL)	114.5 ± 3.3	123.0 ± 26.7	0.06
Homeostatic model for insulin Resistance (HOMA-IR)	9.4 ± 14.1	7.9 ± 5.7	0.47
Lipids			
LDL (mg/dL)	108.9 ± 34.1	107.5 ± 28.1	1.00
HDL (mg/dL)	38.6 ± 8.9	36.3 ± 7.0	0.25
Triglycerides (mg/dL)	139.9 ± 96.3	150.3 ± 99.4	0.50
Total cholesterol (mg/dL)	173.8 ± 38.3	173.8 ± 40.8	0.78
Anthropometrics			
BMI (kg/m^2^)	32.6 ± 5.2	34.2 ± 5.6	0.11
Birthweight (lbs)	7.31 ± 1.79	7.03 ± 1.50	0.33
Waist circumference (cm)	104.8 ± 14.4	109.6 ± 13.9	0.07

*Based on biopsy alone.

Metabolic correlates at baseline were also compared between those that improved and those that did not. Markers of glucose metabolism tended to be lower in those that improved including fasting glucose levels (86.8 ± 9.7 versus 90.2 ± 9.3 ml/dL; p = 0.04) and the 2-h glucose tolerance test (114.5 ± 3.3 versus 123.0 ± 26.7 ml/dL; p = 0.06).

### Characteristics associated with LTL at baseline

Demographic factors associated with LTL at baseline included an inverse association with age (rho = − 0.24; p < 0.001; Table 3) with the shortest LTL in children > 13 years (1.2 ± 0.22 T/S; p = 0.04; Table 3). Metabolic factors associated with LTL also included an inverse association with waist circumference (rho = − 0.20; p = 0.01), BMI (rho = − 0.23, p < 0.01) and 2-h oral glucose tolerance test although results neared statistical significance (rho = − 0.14; p = − 0.09). Diastolic blood pressure also neared statistical significance for an inverse association (rho = − 0.15; p = 0.07).

**Table 3:** Leukocyte telomere length in relation to baseline characteristics.

	Leukocyte telomere length (T/S ratio) (mean ± SD or correlation coefficient (rho))	p value
	Population mean: 1.33 ± 0.23 T/S	
TONIC treatment group		
Placebo	1.35 ± 0.26	1.0
Metformin	1.30 ± 0.21	0.29
Vitamin E	1.34 ± 0.22	0.77
Demographics		
Sex		
Male	1.32 ± 0.26	0.62
Female	1.35 ± 0.26	
Age at enrolment (years)	− 0.24	< 0.001
Age category (years)		
< 10	1.42 ± 0.18	
10–13	1.37 ± 0.25	0.46
> 13	1.28 ± 0.22	0.04
Mexican origin		
No	1.31 ± 0.22	1.0
Yes	1.35 ± 0.24	0.37
Metabolic health at baseline		
Anthropometrics		
Waist circumference (cm)	− 0.20	0.01
Body mass index	− 0.23	< 0.01
Birthweight (lbs)	− 0.03	0.69
Glucose control		
Insulin (mcU/mL)	− 0.04	0.62
Oral glucose tolerance test (OGTT), Glucose, 2 h (mg/dL)	− 0.14	0.09
Glucose, fasting	− 0.10	0.21
HOMA-IR	− 0.09	0.27
HOMA-IR		
≥ 3.99	1.32 ± 0.23	0.28
< 3.99	1.37 ± 0.25	
Blood pressure		
Systolic blood pressure (mmHg)	− 0.06	0.44
Diastolic blood pressure (mmHg)	− 0.15	0.07
Lipids		
LDL (mg/dL)	0.08	0.31
HDL (mg/dL)	0.03	0.70
Triglycerides (mg/dL)	− 0.02	0.80
Total cholesterol (mg/dL)	0.06	0.46
Liver health at baseline		
Liver function tests		
Alanine transaminase (ALT) (U/L)	0.02	0.84
Aspartate aminotransferase (AST) (U/L)	0.07	0.43
Alkaline phosphatase (U/L)	0.14	0.09
Total bilirubin (mg/dL)	− 0.10	0.22
Direct bilirubin (mg/dL)	0.08	0.36
ALT (U/L) quartiles		
< 73	1.35 ± 0.25	1.0
73–90	1.33 ± 0.26	0.69
91–< 145	1.32 ± 0.22	0.27
≥ 145	1.34 ± 0.19	0.84
AST (U/L) quartiles		
< 43	1.34 ± 0.28	1.0
43–55	1.32 ± 0.26	0.69
56–< 78	1.31 ± 0.21	0.63
≥ 78	1.34 ± 0.19	0.90
ANA autoantibody		
Positive	1.29 ± 0.20	0.34
Negative	1.34 ± 0.24	
General liver biopsy findings at baseline		
Steatosis baseline (increasing)	0.07	0.42
Lobular inflammation (increasing)	0.15	0.08
Portal inflammation (increasing)	0.16	0.06
Fibrosis (increasing)	0.10	0.24
Ballooning (increasing)	− 0.04	0.62
Lobular inflammation at baseline		
0–< 2	1.29 ± 0.21	1.0
2–4	1.34 ± 0.25	0.17
> 4	1.47 ± 0.21	0.02
Portal inflammation at baseline		
None	1.25 ± 0.19	1.0
Mild	1.32 ± 0.24	0.30
More than mild	1.41 ± 0.22	0.07
Fibrosis at baseline		
0 (none)	1.28 ± 0.28	1.0
1 (mild-moderate Z3 peri Sinusoidal/portal-periportal)	1.34 ± 0.22	0.27
2 (Z3 periportal)	1.34 ± 0.22	0.29
3 (bridging)	1.36 ± 0.24	0.26
Ballooning at baseline		
0 (none)	1.32 ± 0.24	1.0
1 (few)	1.38 ± 0.25	0.15
2 (many)	1.27 ± 0.18	0.39
Steatosis at baseline		
0–1 (≤ 5–33%)	1.29 ± 0.27	1.00
2 (34–66%)	1.37 ± 0.20	0.12
3 (> 66%)	1.33 ± 0.22	0.44
Steatosis location at baseline		
Zone 1-periportal	1.35 ± 0.20	1.0
Zone 3-periportal	1.34 ± 0.26	0.29
Azonal	1.30 ± 0.20	0.12
Panacinar	1.32 ± 0.23	0.21
Any NASH baseline		
No (not NASH)	1.25 ± 0.28	1.0
Yes (borderline or definite NASH)	1.34 ± 0.22	0.07
NASH baseline		
No NASH	1.25 ± 0.28	1.0
Borderline	1.36 ± 0.24	0.08
Definite NASH	1.32 ± 0.20	0.23
NASH activity score (NAS) (ballooning + lobular + steatosis)	0.10	0.25
Hepatic injury/necrosis specific parameters from liver biopsy		
Acidophil bodies		
Rare/absent	1.30 ± 0.23	1.0
Many	1.35 ± 0.23	0.18
Mallory bodies		
Rare/absent	1.33 ± 0.23	1.0
Many	1.18 ± 0.30	0.45
Microgranulomas		
No	1.28 ± 0.20	1.0
Yes	1.33 ± 0.23	0.64
Pigmented macrophages		
Rare/absent	1.24 ± 0.16	1.0
Many	1.33 ± 0.23	0.38
Metabolic associated characteristics from liver biopsy		
Glycogen nuclei		
Rare/absent	1.33 ± 0.25	1.0
Many	1.33 ± 0.22	0.63
Large lipogranulomas		
Rare/absent	1.33 ± 0.24	1.0
Many	1.3 4 ± 0.22	0.32
Megamitochondria		
Rare/absent	1.33 ± 0.23	1.0
Many	1.33 ± 0.22	0.8
Microvesicular steatosis		
Absent	1.33 ± 0.24	1.0
Present	1.32 ± 0.15	0.63

Histologic-specific factors that were associated with LTL included lobular and portal inflammation which positively associated with LTL although results only trended towards statistical significance (rho = 0.15, p = 0.08) and (rho = 0.16, p = 0.09; Table 3). Those with > 4 inflammatory cells in lobular parenchyma at 20 × magnification had longer LTL compared with those with fewer (1.47 ± 0.21 T/S versus 1.29 ± 0.21 T/, p = 0.02). Those with any NASH at baseline (borderline or definite) tended to have longer LTL but the results trended towards statistical significance 1.34 ± 0.22 versus 1.25 ± 0.28 T/S; p = 0.07).

### Multivariable models

**Figure 1:**
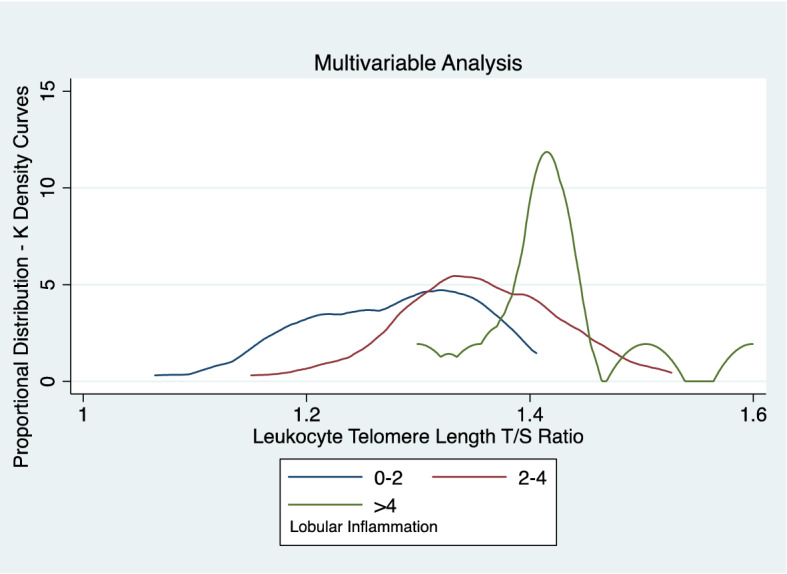
Leukocyte telomere length association with lobular inflammation multivariable analysis.

**Figure 2:**
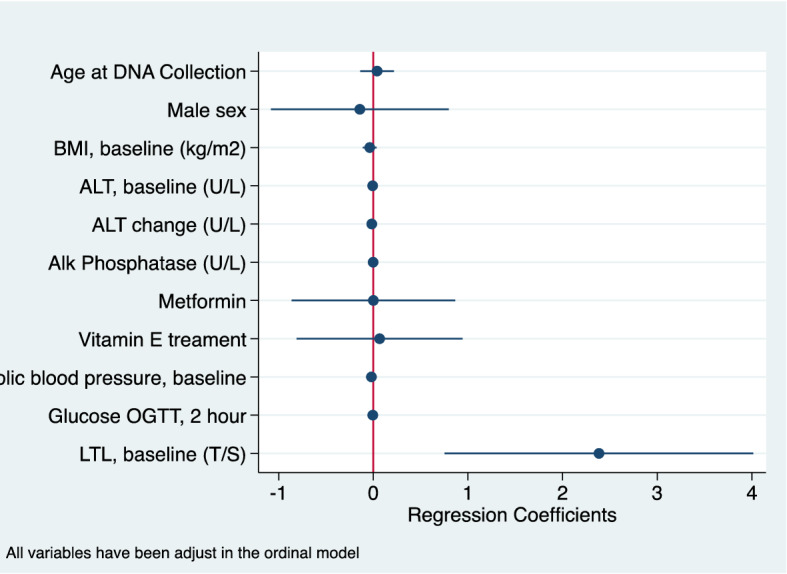
Predictors of lobular worsening at 96 weeks.

In a multivariable model evaluating independent risk factors for NASH improvement at 96 weeks of follow-up, based on biopsy results, including treatment assignment group, sex, LTL, age of enrollment, waist circumference, fasting glucose and alkaline phosphate baseline levels, the only variable that neared statistical significance was fasting glucose level (Odds Ratio (OR), 0.96, 95% CI 0.92–1.01, p = 0.09) in addition to treatment assignment group (vitamin E) (Table 4). A subsequent model that assessed predictors of baseline LTL length including age (years), any NASH at baseline, increasing amounts of portal inflammation, increasing amounts of lobular inflammation, BMI at baseline, diastolic blood pressure (baseline) and glucose oral glucose tolerance test (2 h) suggested that only lobular inflammation was associated with longer LTL at baseline (Coeff = 0.03, 95% CI 006–0.13; p = 0.03) (Table 5; Fig. 1). Another model that evaluated independent predictors of lobular inflammation worsening at 96 weeks including treatment assignment group, age in years, child sex, BMI at baseline, glucose (OGTT) at 2 h, diastolic blood pressure, ALT baseline levels, changes in ALT levels and alkaline phosphate at baseline had LTL at baseline as a positive predictor (Coeff = 2.41, 95% CI 0.78 − 4.04; p < 0.01; Table 6) and ALT at baseline (Coeff = − 0.007, 95% CI − 0.005 to (− )0.0002; Table 6; Fig. 2) and ALT change (Coeff = − 0.02, 95% CI − 0.02 to (− )0.009) as positive predictors (Fig. 2). We ran a similar model evaluating independent predictors of portal inflammation worsening at 96 weeks did not find any statistically significant independent predictors (results not shown).

**Table 4:** Predictors of NASH improvement* at 96 weeks.

Variable	Odds ratio	p value
Metformin Tx group	1.65 (0.60–4.51)	0.33
Vitamin E group	3.53 (1.30–9.58)	0.01
Sex (male)	1.49 (0.53–4.22)	0.45
Leukocyte telomere length (T/S)	0.69 (0.12–3.88)	0.67
Age of enrolment (years)	0.98 (0.79–1.21)	0.82
Waist circumference (cm), baseline	0.98 (0.95–1.01)	0.13
Glucose (fasting), baseline	0.96(0.92–1.01)	0.09
Alkaline Phosphate (baseline)	1.00 (0.29–1.01)	0.12

*Based on biopsy.

**Table 5:** Predictors of leukocyte telomere length at baseline.

Variable	Coefficient	p value
Age (years)	− 0.01 (− 0.03 to 0.006)	0.16
Any NASH at baseline	0.03 (− 0.78 to 0.13)	0.57
Portal inflammation (increasing)	0.05 (− 0.03 to 0.14)	0.24
Lobular inflammation (increasing)	0.03 (0.006–0.13)	0.03
BMI, baseline (increasing)	− 0.005 (− 0.012 to 0.002)	0.15
Diastolic, baseline (increasing)	− 0.002 (− 0.005 to 0.002)	0.27
Glucose 2 h, OGTT	− 0.001 (− 0.003 to 0.0005)	0.20

**Table 6:** Predictors of Lobular Inflammation Worsening at 96 Weeks (n = 131).

Variable	Coefficient	p value
Vitamin E Tx group	0.06 (− 0.82 to 0.93)	0.90
Metformin Tx group	0.005 (− 0.86 to 0.87)	0.99
Sex (male)	− 0.16 (− 1.09 to 0.77)	0.74
Leukocyte telomere length (T/S)	2.41 (0.78–4.04)	< 0.01
Age of DNA collection, yrs	0.08 (− 0.11 to 0.26)	0.43
BMI, baseline	− 0.04 (− 0.11 to 0.03)	0.29
Glucose (2 h), OGGT	− 0.006 (− 0.02 to 0.01)	0.48
Alkaline phosphate (baseline)	− 0.001 (− 0.006 to 0.004)	0.61
Diastolic blood pressure (baseline)	− 0.02 (− 0.05 to 0.01)	0.24
ALT (baseline)	− 0.007 (− 0.005 to (− )0.0002)	0.047
ALT change	− 0.02 (− 0.02 to (− )0.009)	< 0.01

Lastly, we ran a final model of lobular inflammation worsening at 96 weeks but included specifics from the biopsy report that were predictive of lobular improvement in biariate analysis. Specifically, we included presence of acidophil bodies and microvesicular steatosis at baseline (both which were significantly associated with lobular inflammation in univariate analysis). In a multivariable model adjusting for all the previous covariates mentioned in the previous lobular inflammation model, neither were associated with lobular inflammation worsening (results not shown).

## Discussion

This is the first study to evaluate the role of LTL as a predictive biomarker in pediatric patients with NAFLD including a high proportion with NASH. The mean value of 1.33 ± 0.23 T/S units in our population group is longer than that in other studies with children of roughly the same age including a study of Mexican children (1.03 ± 0/74)^18^ and Brazilian ones (1.05 ± 0.42 T/S) in whites and 1.18 ± 0.57 T/S in black or mixed children)^19^. As described below, it is possible that the NAFLD disease process in children activates telomerase or other mechanisms to maintain telomere length, and this process may be indicative of a more severe liver disease process in children. Indeed, NAFLD is often more severe in children than adults with estimates of 18% of children diagnosed with stage 3 fibrosis^20,21^ and the portal-based injury that is typical of NASH in children is likely a better predictor of severe disease than lobular inflammation^22^.

Previous studies have assessed LTL in adults with NASH finding an association between liver fibrosis and shorter LTL^5^ but no associations between other hepatic histological characteristics including inflammation and LTL measurements in contrast with our findings. It is possible that we did not find any association between fibrosis and LTL in our study, as the pattern of hepatic injury is different in children (or potentially the rate of fibrosis progression may be faster in children and this may impact LTL).

As pediatric NAFLD has histological characteristics that differ from adult NAFLD, processes of pathogenesis of pediatric versus adult NAFLD may impact telomeres differentially. In general, pediatric NAFLD is characterized by portal inflammation and fibrosis versus lobular inflammation and hepatocyte ballooning in adult NAFLD^23,24^ with portal inflammation associated with a more severe disease phenotype in both adults and children^22^. In this cohort of pediatric patients, 92.6% of patients had portal inflammation with 12.1% demonstrating “more than mild” portal inflammation. Although the majority also had lobular inflammation, a significant proportion were categorized in the mild range with 43.3% < 2 foci at 20 magnification.

Previous longitudinal studies have found an association between longer LTL in patients with chronic hepatitis B and C infection and increased risk of hepatocellular carcinoma (HCC)^25,26^ particularly earlier in the disease process, versus right before diagnosis when shorter LTLs were more common^26^. A study from China also found longer LTLs among patients with hepatitis B associated- chronic liver disease compared with healthy controls using a case control study design^27^. Longer telomeres may result in elevated cancer risk and potentially other pre-malignant processes by delaying paths of cellular senescence and apoptosis, resulting in prolonged cellular exposure to genetic and environmental insults increasing risk for genetic lesions^27^. Telomerase is more commonly detected in HCC tissue compared with patients with chronic liver disease, but chronic liver disease patients have evidence of telomerase activity in approximately 10% of cases^28^. Telomere maintenance processes in HCC development take heterogenous forms resulting in different pathways and aggression levels^29^. It is possible that the pediatric form of NAFLD may resemble specific hepatic pathological processes more commonly seen in adults, particularly adults earlier in a malignant disease process.

As shorter LTL is associated with systemic oxidative stress exposure and severity of metabolic disease, it is possible that our finding of longer LTL with increasing amounts of lobular inflammation may suggest alternate pathways, including potential future risk for cancer in these children^30,31^. Long-term longitudinal studies of pediatric patients with NAFLD have not yet been conducted. As such, our natural history understanding of how NALFD changes from pediatric patients to adult ones is limited. Lobular inflammation is a more common component of adult NAFLD and worsening of lobular inflammation as well as longer LTL may be suggestive of worsening disease.

Limitations of this study include the absence of reference ranges for LTL. As such, although we indicate that the children in this study may have longer LTL than those from similar population groups, reference ranges would help standardize comparisons between groups. There are differences in LTL based on lab techniques and other population factors that we are unable to account for and as such comparisons must take these limitations into consideration. Finally, future studies should collect telomere length from liver cells in patients with NAFLD including hepatocytes, cholangiocytes, Kupffer and stellate cells to better understand how histological changes correlate with telomere length changes including those from both liver cells and leukocytes.

## Data Availability

Access to data will be provided based on request to the corresponding author and the NASH CRN.
